# Whole-exome sequencing identifies somatic mutations and intratumor heterogeneity in inflammatory breast cancer

**DOI:** 10.1038/s41523-021-00278-w

**Published:** 2021-06-01

**Authors:** Rui Luo, Weelic Chong, Qiang Wei, Zhenchao Zhang, Chun Wang, Zhong Ye, Maysa M. Abu-Khalaf, Daniel P. Silver, Robert T. Stapp, Wei Jiang, Ronald E. Myers, Bingshan Li, Massimo Cristofanilli, Hushan Yang

**Affiliations:** 1 Department of Medical Oncology, Sidney Kimmel Cancer Center, Thomas Jefferson University, Philadelphia, PA USA; 2 Department of Molecular Physiology and Biophysics, Vanderbilt University, Nashville, TN USA; 3 Department of Pathology, Sidney Kimmel Cancer Center, Thomas Jefferson University, Philadelphia, PA USA; 4 Division of Hematology Oncology, Feinberg School of Medicine, Northwestern University, Chicago, IL USA

**Keywords:** Cancer genomics, Tumour heterogeneity, Breast cancer

## Abstract

Inflammatory breast cancer (IBC) is the most aggressive form of breast cancer. Although it is a rare subtype, IBC is responsible for roughly 10% of breast cancer deaths. In order to obtain a better understanding of the genomic landscape and intratumor heterogeneity (ITH) in IBC, we conducted whole-exome sequencing of 16 tissue samples (12 tumor and four normal samples) from six hormone-receptor-positive IBC patients, analyzed somatic mutations and copy number aberrations, and inferred subclonal structures to demonstrate ITH. Our results showed that *KMT2C* was the most frequently mutated gene (42%, 5/12 samples), followed by *HECTD1*, *LAMA3*, *FLG2*, *UGT2B4*, *STK33*, *BRCA2*, *ACP4*, *PIK3CA*, and *DNAH8* (all nine genes tied at 33% frequency, 4/12 samples). Our data indicated that *PTEN* and *FBXW7* mutations may be considered driver gene mutations for IBC. We identified various subclonal structures and different levels of ITH between IBC patients, and mutations in the genes *EIF4G3*, *IL12RB2*, and *PDE4B* may potentially generate ITH in IBC.

## Introduction

Inflammatory breast cancer (IBC) is an aggressive form of breast cancer defined by the rapid onset of inflammatory signs (such as erythema, edema, warmth, and induration) involving more than one-third of the breast^1–3^. IBC accounts for 1–6% of breast cancer cases^2,4,5^ yet causes roughly 10% of breast cancer deaths^6,7^. The prognosis in patients with IBC is worse than in non-IBC, with the 3‐year survival rate for IBC patients far lower (around 40%) than patients with other types of breast carcinoma (around 85%)^5,8^. Although treatment approaches based on hormone-receptor (HR) or HER2 status are available, there are no treatments that are specifically recommended for tumors with an IBC phenotype. The scarcity of data from IBC patients and the poor understanding at the molecular level has hindered the development of specific therapeutic interventions. In order to develop potential IBC-specific targeted therapies, obtaining more genomic information is crucial.

Intratumor heterogeneity (ITH) arises from heritable and stochastic genetic and epigenetic changes, as well as environmental variations within the tumor^9^. Since tumors with ITH have subclones with distinct mutations that may relate to cancer-specific phenotypes, ITH is intricately related to cancer progression, resistance to therapy, and recurrences^10^. It is clear that a better understanding of ITH is very important to the development of genome-informed precision medicine^11^.

The rapidly evolving technology of next-generation sequencing (NGS) has made it possible to analyze genomic characteristics of tumor samples at an unprecedented speed. Since 2015, eight NGS-based studies on IBC tumors have been published. Among them, six out of eight used targeted sequencing^12–18^, and two conducted whole-exome sequencing (WES)^19,20^. These studies reported frequently mutated genes in IBC, such as *TP53* (43–75%), *PIK3CA* (13–42%), *BRCA2* (13–26%), *ARID1A* (10–21%), *RB1* (11–16%), and *PTEN* (11–15%)^12–18^. Frequent *HER3* hotspot mutations were also found in IBC tumors and cell line studies confirmed a role for mutant *HER3* in IBC cell proliferation^15^. Frequent genomic alterations in the PI3K/AKT/mTOR pathway have been seen^15^, and somatic activation of this pathway (i.e., *PIK3CA* activating mutation or gain^14^, *ERBB2* activating mutation, *PTEN* deletion, *AKT1* activating mutation) was significantly associated with shorter progression-free survival (PFS) in trastuzumab-naïve HER2-positive IBC patients^19^. However, most of these studies were based on targeted sequencing panels, and none of them provided information for intratumor subclonal structures or evaluated ITH.

In the current study, we performed whole-exome sequencing in 16 tissue samples (12 tumor and 4 normal samples) from six IBC patients to obtain a comprehensive understanding of the IBC genomic landscape. Based on the mutation calls and somatic copy number alterations, we characterized ITH and subclonal structures, identified primary and secondary driver genes for the tumor and subclone formation, which could shed light on potential new treatment strategies for IBC.

## Results

### Patient and sample description

Clinical and pathological information of the six IBC patients (P1–P6) are provided in Supplementary Table 1. The median age at sample collection time was 56 years (ranging from 36 to 72 years). All six patients had HR+ tumors, with 5/6 (83.3%) patients having estrogen-receptor-positive (ER+) tumors, and the other had a progesterone-receptor positive (PR+) tumor. By only considering HR+ IBC tumors, our study eliminated additional confounding introduced by differences in HR subtypes seen in previous studies. Details of the tumor and normal tissue samples obtained from the six IBC patients are found in Supplementary Table 2. The samples from P2 were obtained from an incisional biopsy, which limited the volume of tissue obtained, and these samples were subsequently found to be insufficient for conducting subclone identification.

### Sequencing quality validation

We achieved a mean sequencing depth of ~170× (ranging from 133 to 210×, Supplementary Table 3), with mapping rates exceeding 99% in all 16 samples. After stringent filtering criteria (see Methods), we obtained a total of 1477 somatic mutations. We called 293, 15, 261, 120, 495, and 293 somatic mutations, respectively, in patients P1–P6 (Supplementary Data 1). Four of the six patients (P1, P2, P4, and P6) had matched normal samples, allowing us to validate the stringency of our mutation calling pipeline (see Methods). We identified artifactual mutations in one, six, one, and four instances, respectively, in patients P1, P2, P4, and P6. Artifactual mutations in normal samples also had much lower allele frequencies (AFs) and tended to be obtained at lower depths compared to tumor mutation calls, which indicated that FFPE-induced artifacts had negligible effects to the data presented in our study (Supplementary Fig. 1).

### Somatic mutation identification

We used a somatic mutation classification system as previously described^21^. Five of six patients exhibited mutational signatures characterized predominantly by C > T transitions, with the sixth patient P6 showing a mix of C > G and C > T transitions (Supplementary Fig. 2). These results were consistent with previous reports for breast cancer, which have also found C > T transitions to constitute the majority of somatic mutations^21,22^.

**Fig. 1:**
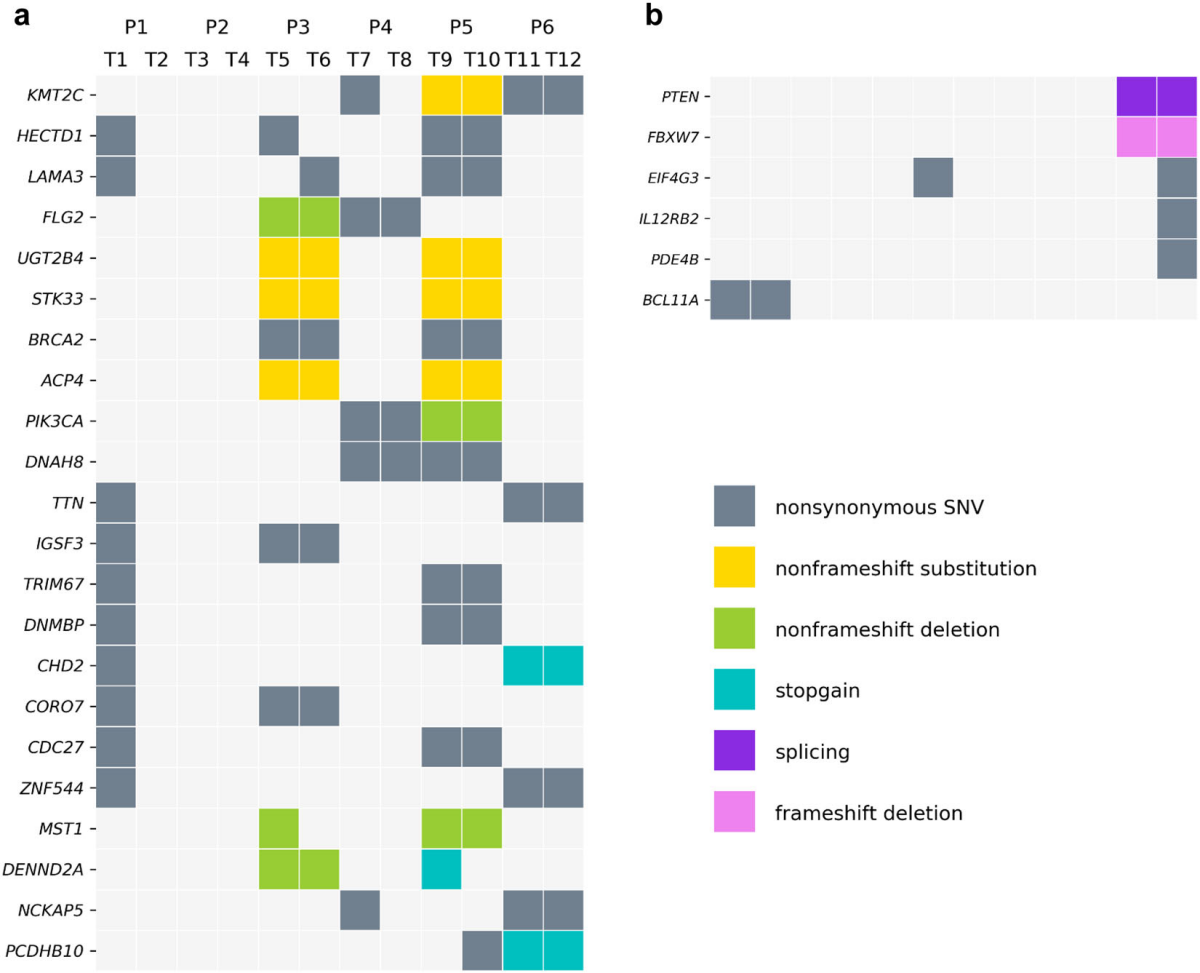
Somatic mutation profile for 12 tumor samples of six patients. **a** The most frequently mutated genes are shown in a heatmap, with the columns representing the 12 tumor samples from six patients, and the rows representing genes. The different colors indicate the type of mutation as indicated in the figure. **b** Heatmap of mutations in selected genes known to be involved in cancer pathogenesis or progression.

In total, we found 787 mutated genes from the 12 tumor samples in six patients. In these samples, *KMT2C* was the most frequently mutated gene (5/12 samples, 42%). Nine mutated genes were found in four samples (33%, including *HECTD1*, *LAMA3*, *FLG2*, *UGT2B4*, *STK33*, *BRCA2*, *ACP4*, *PIK3CA*, and *DNAH8*), and 12 genes were mutated in three different samples (25%, including *TTN*, *IGSF3*, *TRIM67*, *DNMBP*, *CHD2*, *CORO7*, *CDC27*, *ZNF544*, *MST1*, *DENND2A*, *NCKAP5*, and *PCDHB10*). Figure 1a shows the 22 most frequently mutated genes. In addition, mutations in 244 genes were found in two tumor samples, with the remaining gene mutations (in 521 genes) private to single tumor samples.

We also analyzed the gene mutations at the patient-level. *KMT2C*, *HECTD1*, and *LAMA3* were the most frequently mutated genes as they were shared by three of six patients (50%). Histone methyltransferase *KMT2C* is a tumor suppressor gene reported to be a driver gene for breast cancer^23,24^. There were 57 mutated genes identified within two patients (2/6, 33%), and the rest of the mutated genes were not common to multiple patients. All counted mutations were nonsynonymous (i.e., frameshift/non-frameshift indel, stop-gain/stop-loss, splicing, or nonsynonymous SNV).

### Copy number aberration (CNA) inference

**Fig. 2:**
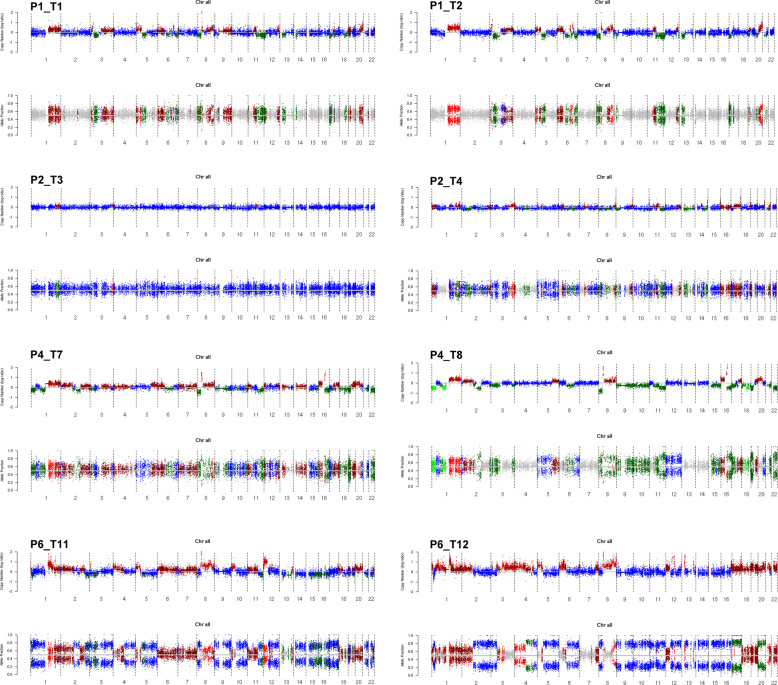
Graphical representations of copy number alterations (CNAs) and allelic fractions (AFs) from two tumors per patient. Two tumors are obtained from each patient, represented on the left and right of the figure. Data from four patients with matched normal samples are available, and four sets of CNAs and AFs plots are presented. Within each set, the CNA plot is shown above the AF plot. For the CNA plot, the *y*-axis is the log2 ratio of the copy numbers of tumor to normal sample, both normalized for read depth. Points close to 0 (midline) represent no change in copy number, above 0 are copy gains, and below 0 are deletions. Regions are colored as follows: bright green (homozygous deletion), green (hemizygous deletion), blue (diploid heterozygous or copy-neutral loss of heterozygosity), dark red (copy number gain), and red (allele-specific CNA, unbalanced CNA, balanced CNA). The *x*-axis represents chromosomes. For the AF plot, the *y*-axis is the frequency of the reference allele in a germline heterozygous SNV, and the expected heterozygous frequency of 0.5 is the midline. Data points close to 1 represent homozygous reference, and data points close to 0 represent the homozygous nonreference base. Regions are colored as follows: gray (heterozygous, or balanced CNA), bright green (homozygous deletion), green (hemizygous deletion), blue (copy-neutral loss of heterozygosity), dark red (copy number gain), and red (allele-specific CNA, unbalanced CNA). The *x*-axis represents chromosomes.

We obtained ~25,000 germline variants in each patient with matched normal samples (P1, P2, P4, and P6). We used TITAN, a probabilistic model that simultaneously infers CNA and loss of heterozygosity (LOH) segments from read depth and digital allele ratios at germline heterozygous SNP loci across the exome from tumor WES data^25^. Figure 2 shows the profiles of CNAs for the four patients with matched normal samples. We observed that patient P2 had a relatively low tumor cell fraction. Patient P6 had the best sample quality and showed extensive LOH.

### Subclone identification

**Fig. 3:**
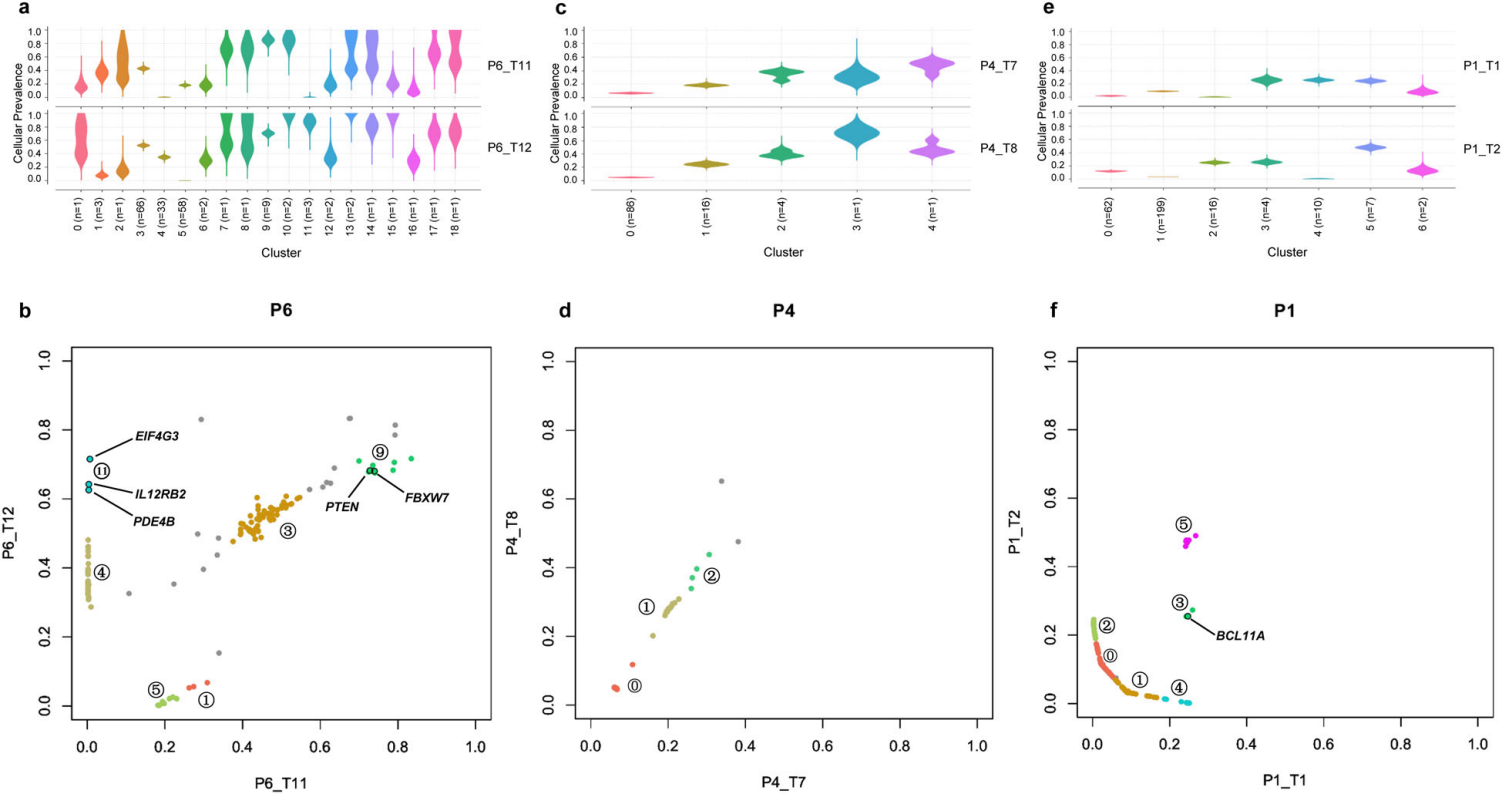
Cancer cell fractions (CCFs) of subclones and mutations for patients P6, P4 and P1. We computed the CCF for each mutation found in the tumor samples from patients P6, P4, and P1. Hierarchical clustering of mutations is then performed to obtain putative subclones. **a, c, e** Subclone CCF density figure shown as violin plots. The *x*-axis represents subclone clusters, and the n value shows the number of mutations in each subclone. Each mutation has two CCF values (as they may be detected in both tumor samples). The *y*-axis represents the density of CCFs for each subclone. **b, d, f** Scatter plot of CCFs for each patient. Each point represents a mutation. The *x*- and *y*-axis represent the CCFs of a mutation for each of two tumor samples in a patient. Mutations belonging to clusters *n* ≥ 3 are shown in their corresponding cluster colors in the violin plot. Mutations belonging to clusters *n* < 3 are outliers (shown in light grey) and not clustered. **a** 19 PyClone inferred subclone clusters in P6. **b** CCF relationship for P6. Subclone 1, 3, 4, 5, 9, and 11 in **a** are marked. *PTEN*, *FBXW7*, *EIF4G3*, *IL12RB2*, and *PDE4B* mutations are labelled with arrows. **c** Five PyClone inferred subclone clusters in P4. **d** CCF relationship for P4. Subclone 0, 1, and 2 in **c** are marked. **e** Seven PyClone inferred subclone clusters in P1. **f** CCF relationship for P1. Subclone 0, 1, 2, 3, 4, and 5 in **e** are marked. *BCL11A* mutation is labelled with arrows.

Using CNA information, we conducted PyClone analysis to estimate cancer cell fractions (CCFs) of all mutations and then assigned each mutation to different subclones (see Methods). For each patient, we obtained the subclone CCF density (represented as violin plots) and plotted CCFs in one tumor sample against the other tumor sample (as a scatter plot) (Fig. 3). Major subclones from the density plots are labelled in the same color in the scatter plot.

**Fig. 4:**
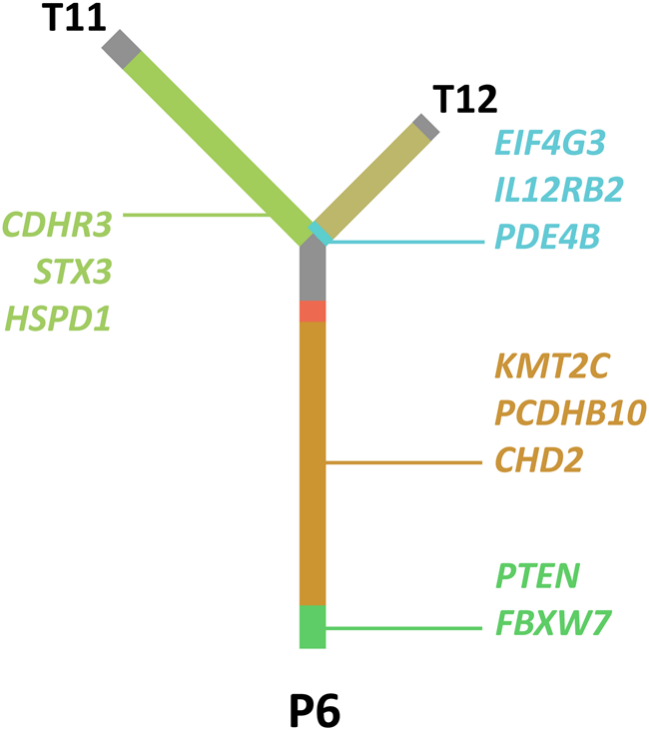
The Phylogenetic tree of tumor samples from patient P6. The trunk of the tree represents clusters of mutations that are common to both tumor samples, likely representing truncal mutations. The lengths of the trunk and the branches of the tree are proportional to the number of corresponding mutations. Major subclones are labelled in the same color as in Fig. 3b. Gene labels refer to mutations in genes that are identified in T11 only, T12 only, or common to both tumors. The gene labels are nonexhaustive.

For patient P6 (Fig. 3a, b), we observed six distinct subclones with different cluster CCFs. Subclone 4, 5, and 11 all had very low subclone CCFs in one of the two samples (but high CCFs in the other sample), indicating clear ITH. Subclone 9 had cluster CCFs of greater than 0.7 in both samples, suggesting a high possibility of this subclone containing driver genes. This subclone also contained mutations in *PTEN* and *FBXW7*, both tumor suppressor genes previously reported^26,27^ as driver genes for breast cancer. Subclone 11 contained *EIF4G3*, *IL12RB2*, and *PDE4B* mutations, and all three mutations had zero allele frequencies in the tumor sample P6_T11, indicating the possibility of secondary driver genes for this subclone. We used Integrative Genomics Viewer (IGV) to check and confirm that the high CCFs of these genes were not caused by duplication. *EIF4G3*, *IL12RB2*, and *PDE4B* genes are all located in chromosome 1. Figure 4 shows the phylogenetic tree for P6. In order to further explore the relationships between different subclones in patient P6, we constructed the subclonal architecture based on cluster CCFs (see Methods). Supplementary Figure 3 depicts the deduced linear and/or branching relationships of subclones in P6. For example, in architecture c (one of the four possible subclonal architectures of sample T11), subclone 9 represented the subclonal trunk mutations, with subclone 3, 1, and 5 all derived from it (i.e., they were all linear in relationship to subclone 9). Subclone 5 was derived from subclone 3, but subclone 3 and 1 occupied different subpopulations of cells (i.e., subclones 3 and 1 were diverging branches).

Three major subclones were found in patient P4 (Fig. 3c, d), and their subclone CCFs had little differences between the two samples, indicating high similarity between tumor samples from P4.

Six major subclones were identified in patient P1 (Fig. 3e, f). Most of the mutations had CCFs below 0.2 (subclone 0 and 1), while subclone 2 and 4 reflected ITH. Also, a mutation of the driver gene *BCL11A*^28^ was found in subclone 3. Figure 1b shows these important functional genes.

## Discussion

To obtain a better understanding of the genomic alterations and ITH in inflammatory breast cancer, we applied WES to matched normal and tumor samples of IBC patients. Herein, we report the frequently mutated genes, varying levels of ITH, subclonal structures and possible driver genes in different patients. Our study is one of the few attempts using WES to analyze IBC^19^ and investigate ITH with subclonal structures in IBC.

Previous studies have reported the proportion of positive receptors in IBC tumors. The prevalence of overexpressed or amplified HER2 was about 40% (compared with 25% in non-IBCs), and the prevalence of HR positivity is lower, about 30% (compared with 60–80% in non-IBCs)^29^. The HR+ percentage of IBC tumors in recent NGS-based studies was about 39% (ranging from 29 to 54%)^12–18^. However, since HR+ IBC patients tend to have worse clinical outcomes than HR+ non-IBC patients^29^, this study sought to explore the genomic landscape of HR+ IBC tumors. This strategy also prevents potential confounding effects from HR subtypes, in contrast to previous IBC studies.

We found a frequently mutated gene *KMT2C*, which has been reported as frequently altered in other IBC^30^ (15% mutation rate) and non-IBC^16^ cases (11% mutation rate). As a reported driver gene, *KMT2C* had the highest genetic mutation rate among histone methyltransferases in breast cancer and was most frequently mutated in Luminal A breast cancer^31^. Previous works demonstrated that *KMT2C* mediated ER-independent growth of HR+ breast cancer cell lines^24,32^ and *KMT2C* loss promoted hormone-independent ER+ breast cancer cell proliferation^32^. Thus, the HR positivity of our samples could be an important factor for the enrichment of *KMT2C* mutation found in our study (a 42% mutation rate). The deletion of *KMT2C* is significantly associated with shorter PFS^32^, and amplification/gain of this gene was significantly associated with longer survival, compared with patients who had no change in copy number^32^.

In patient P6, *PTEN* and *FBXW7* mutations were detected at high CCFs, thus they may be driver mutations for this patient. The lipid phosphatase *PTEN* is a major negative regulator of the PI3K/Akt/mammalian target of rapamycin (mTOR) pathway^26^. PI3K inhibitors, such as alpelisib, have been approved for treatment of *PIK3CA*-mutant ER+ breast cancers^33^. Everolimus (a rapamycin analog and an inhibitor of the mTOR pathway) has also been approved for ER+ breast cancer^34^. *FBXW7* is a critical tumor suppressor, which controls the proteasome-mediated degradation of mTOR^27^. Human breast cancer cell lines harboring deletions or mutations in *FBXW7* are particularly sensitive to rapamycin treatment^27^. Finally, breast cancer patients with lower *FBXW7* mRNA expression had poorer survival^35^.

Also in patient P6, *EIF4G3*, *IL12RB2,* and *PDE4B* mutations only occurred in sample T12 and formed a subclone with relatively high CCF (>0.6). This was an interesting finding as it indicated that this subclone was newly generated only in a specific area of the tumor. These genes seemed to have a strong positive selection in specific environment and conditions, as well as a potential to drive secondary tumor progression.

Phosphodiesterase type IV (PDE4) degrades the intracellular second messenger cyclic AMP in many cell types. As PDE4s regulate many active processes such as immune cell proliferation and inflammatory mediators releasing, PDE4 inhibitors are potent inhibitors of inflammation, and they have been approved for the treatment of many inflammatory diseases including asthma, arthritis and chronic obstructive pulmonary disease^36,37^. Previous works showed that *PDE4B* is a potential therapeutic target as well as prognostic molecular marker in colorectal cancer^38,39^. Further study is needed to investigate if *PDE4B* could also be a therapeutic target or marker for IBC patients.

*IL12RB2*, which encodes for one chain of the interleukin-12 (IL-12) receptor, is involved in several inflammatory diseases^40^. IL-12 is a heterodimeric proinflammatory cytokine. Overexpression of IL-12 can cause persistent inflammation^41^, thus contributing to the aggressive nature of IBC^29^. Genetic polymorphisms in *IL12RB2* are associated with increased risk of chronic inflammatory disease^42^. Also, hyperactivation of the IL-6 pathway is frequently observed in IBC, and associated with poor prognosis^29^. In our samples, we observed a high percentage of tumor cells harboring *IL12RB2* mutations (i.e., high CCF), though it remains unclear whether the *IL12RB2* mutations play any functional roles in influencing the inflammatory pathways.

The presence of ITH in patients with IBC or other cancers indicates that an individual tissue biopsy may be insufficient to evaluate the genomic profile of an entire tumor, which could introduce bias in the selection of personalized therapies. For example, the gene coding for the estrogen receptor, *ESR1*, is often found to be mutated in metastatic ER+ breast cancers previously treated with estrogen therapy^43^. The high *ESR1* mutational prevalence in previously treated tumors, juxtaposed with the rarity of *ESR1* mutations in treatment-naïve primary tumors, suggest the development of resistance subclones during treatment, and thus has raised much interest in understanding ITH^43^. Furthermore, several landmarks of disease progression in breast cancer, such as resistance to chemotherapy and metastases, arose within detectable subclones in the primary tumor^44^. These findings highlight the importance of subclonal structure analysis.

In this study, conducting WES on multiple samples from each IBC tumor allowed us to investigate many more genes than using targeted sequencing, and thus we were able to identify specific subclonal structures and ITH. However, the main limitation of our study is the small sample size. Given the rarity of IBC, many genomic studies on this disease subtype face challenges in acquiring enough samples. In this study, the tumor tissues without matched normal specimens further reduced the number of available samples. Moreover, although we demonstrated extensive ITH in HR+ IBC, the limited sample size prevented us from reaching more definitive conclusions on the role of clonal expansion in IBC. One interesting aspect is the genomic level comparison between IBCs and non-IBCs, which remains underexplored. A previous study using immunohistochemistry suggested overexpression of E-cadherin to be a key difference^45^, but large-scale nonbiased approaches are also needed. Further research comparing IBC and non-IBC samples with matched clinical characteristics may uncover the genomic origin of IBC. To definitively answer the effects of clonal expansion on the inflammatory phenotype of IBC, non-IBC patients who have inflammatory recurrence during follow-up could be enrolled, to compare primary non-IBC tumor tissues with tumor tissue at recurrence. Another limitation of this study is the lack of information regarding treatments prior to sample collection for some patients. Patient P6 received chemotherapy before sample collection, which could possibly influence the genomic signature and result in significant ITH.

In conclusion, we conducted WES on multiple samples of human IBC tumors with matched normal samples, and our results revealed the high frequency and diversity of somatic mutations, subclonal structures, differing levels of ITH, and potential driver genes in IBC patients. These findings encourage future studies and clinical trials for developing targeted therapies that could benefit IBC patients.

## Methods

### Patient samples

Sixteen samples were collected from six IBC patients, including 12 tumors (two from each patient) and 4 matched normal samples (in four out of six patients). The six patients P1–P6 were enrolled between 1993 and 2012. This study was based on detecting archived tissue samples and reviewing archived medical/pathologic reports. Patient consent was waived by the Institutional Review Board of the Office of Human Research at Thomas Jefferson University under an approved protocol.

### Identification of molecular subtype

Immunohistochemical (IHC) staining of paraffin-embedded tissue sections with monoclonal antibodies were used to determine patients’ ER and PR status as part of a routine diagnostic procedure. HR status was positive if the patients were either ER or PR positive. HER2 status was also determined by IHC staining following standard guidelines at the time of diagnosis. The FDA approved DAKO guidelines were used for scoring patient P5 (2004)^46,47^. The 2007 ASCO/CAP guideline^48^ was used for patient P6 (2012). There were no standard guidelines before the FDA approval, therefore we matched the old scoring systems^49,50^ with modern standards for those early patients (P1–P4). The percentage of ER- and PR-positive cells and HER2 status scores were obtained from pathological reports and shown in Supplementary Table 1.

### DNA extraction and WES

For all tumor samples, IBC diagnosis was confirmed by two independent pathologists and the tumor regions were macro-dissected under a microscope. For each sample, we extracted total DNA from approximately ten 14-um sections of formalin-fixed, paraffin-embedded (FFPE) blocks (tissue surface area, 100–150 mm^2^) using the AllPrep DNA/RNA FFPE kit (Qiagen), with a protocol we empirically optimized. The AllPrep kit is well-validated on long-term preserved FFPE samples^51,52^. Before library construction, all DNA samples were assessed using a NanoDrop spectrophotometer for OD 260/280 and OD 260/230, a Qubit fluorometer for concentration, and a 2100 Bioanalyzer (Agilent) for peak analysis. We then performed WES (using SeqCap EZ Exome 2.0 kit from Nimblegen for library construction) on Illumina HiSeq 2000 paired-end sequencing system.

The human genome GRCh37 was used as a reference and the raw reads were aligned using BWA-0.7.17^53^. The BAM files were generated through samtools-1.9, then further processed through duplicates marking, Base Quality Score Recalibration (BQSR), gVCF generating, joint genotyping and Variant Quality Score Recalibration (VQSR) by GATK-4.1.0.0^54^. The sequencing quality assessment was evaluated by QPLOT^55^.

### Mutation calling and quality control

Based on the best practice procedures for sequencing alignment and quality control^56^, somatic mutations were called by MuTect2 using genomic references from the Broad Institute^57^. We created a Panel of Normals (PoN) by aggregating all the normal samples so that we could remove common germline variants as well as commonly noisy sites (e.g., mapping artifacts or other somewhat random but systematic artifacts of sequencing). This PoN also served as the normal sample for P3 and P5 since they did not have matched normal samples for somatic calling. We applied the default filter to conservatively select somatic calls with confidence.

Final mutation calls were selected through a stringent filtering process and functionally annotated by ANNOVAR^58^.

We applied the following filtering criteria for somatic mutation calling: (1) read depth > 25; (2) mutant AF > 0.05 in tumor samples; (3) corresponding allele frequency <0.01 in matched normal samples (if present); (4) mutations listed in 1000 Genomes Project^59^ or Exome Sequencing Project^60^ removed.

The following filtering criteria were applied for germline variant calling: (1) read depth ≥ 50; (2) genotype quality score ≥ 30; (3) allele fractions ≥0.3 and ≤0.7; (4) multiple-allele variants removed; (5) variant quality score recalibration (VQSR) ≤ 97.00; (6) variants in segmental duplication removed^61^.

We validated the quality of our somatic mutation calls using methods that we have previously established^61^. Briefly, when running Mutect2 in patients with matched normal samples (P1, P2, P4, and P6), we performed the same pipeline and filtering criteria but switched the normal and tumor samples. The mutation calls that passed the criteria are declared as artifactual mutations. If there were major artifacts in FFPE samples, we would be able to call artifactual mutations in matched normal samples since they were also FFPE samples.

### Copy number aberration (CNA) inference

CNAs were inferred using TITAN-1.26.0^25^ based on the called germline heterozygous variants information. CNA analysis was only performed on tumor samples with matched normal.

First, we used HMMcopy-0.99.0^62^ to count the number of reads in nonoverlapping windows of 10 kb directly from BAM files. Then we obtained corrected read depth using mappability and GC content. CNAs were inferred by the ratios of tumor/normal, mutant/reference depths at the germline heterozygous variants sites. We set the maximum copy number to 5 and the number of clonal clusters to 2 in the TITAN settings.

### Subclone inference

Finally, we inferred subclones using PyClone-0.13.1^63^ based on the obtained CNA information. PyClone is a hierarchical Bayes statistical model that uses the measurement of allelic prevalence in deep sequencing data to estimate the proportion of tumor cells harboring a mutation (referred to herein as ‘cancer cell fraction’ (CCF))^63^. We first computed the CCF for each mutation, and then performed hierarchical clustering to assign each mutation to one cluster (subclone).

In the PyClone settings, the number of iterations was set to 50,000 and the density model was chosen to be Beta Binomial emission. In order to obtain a better result, we optimized the input parameters and custom-built the yaml mutations files.

### Construction of subclonal architecture

We deduced linear and/or branching evolutionary relationships of all subclones in patient P6 based on their cluster CCFs using established methods^61^. A linear relationship between two subclones would indicate that the one with smaller CCF was derived from the one with larger CCF, suggesting that the mutations in the derived subclone occurred later in the same ancestral cells, which already carried the mutations in the larger subclone. A branching relationship between two subclones would indicate that the mutations in each of the subclones occurred in different ancestral cells and the subclones occupied different portions of the tumor cells.

### Reporting summary

Further information on research design is available in the Nature Research Reporting Summary linked to this article.

## Supplementary information

Supplementary Information

Supplementary Data 1

Reporting Summary

## Data Availability

The data generated and analyzed during this study are described in the following data record: 10.6084/m9.figshare.14538252^64^. Release of full genetic sequencing data was not included in the IRB protocol. Thus, only sequencing data related to this paper have been released, and these data have been deposited in NCBI Sequence Read Archive (SRA) with the accession code https://identifiers.org/ncbi/bioproject:PRJNA713359^65^. Additional files underlying the figures and supplementary figures are available as part of the figshare data record.

## Code availability

The codes for Fig. 1 and Fig. 3, which were written in Python and R languages are available upon request.
