# Supplementary material for: Whole-exome sequencing identifies somatic mutations and intratumor heterogeneity in inflammatory breast cancer
Source: NPJ Breast Cancer. 2021 Jun 1;7:72. doi: 10.1038/s41523-021-00278-w (PMC8169683; doi:10.1038/s41523-021-00278-w)
Supplement: Supplementary file 1 — Supplementary Information [file 41523_2021_278_MOESM1_ESM.pdf]

Supplementary information

Supplementary Figure 1. Plots of allele frequency over sequencing depth for mutations identified in tumor and matched normal samples.

Supplementary Figure 2. The distribution of somatic mutations types for each patient.

Supplementary Figure 3. Subclonal architecture for patient P6.

Supplementary Table 1. Clinical and pathological information of the six IBC patients

Supplementary Table 2. Details of the tumor and normal tissue samples in the six IBC patients

Supplementary Table 3. QPLOT results, including mean depth, insert size and mapping rate

Supplementary Data 1. List of all mutations identified in patients P1-P6

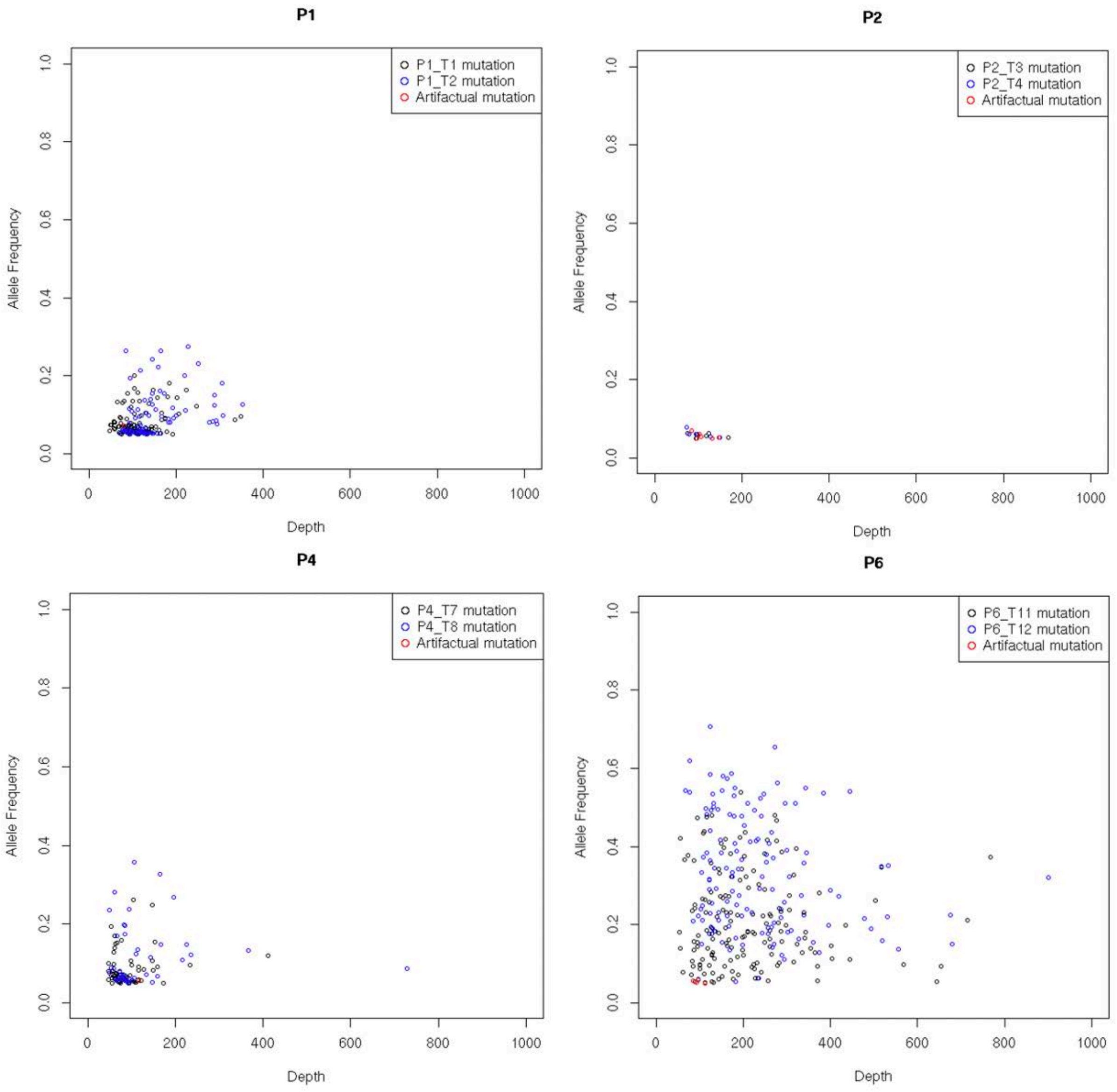

Supplementary Figure 1. Plots of allele frequency over sequencing depth for mutations identified in tumor and matched normal samples.

Four patients had matched normal samples (P1, P2, P4 and P6). We obtained two tumor samples from each patient, with mutations identified from the two samples represented as black or blue circles respectively. Patient 6 has many mutations, including several detected at moderate depths with high allele frequencies, which are indicative of driver mutations. Artifactual mutation calls are represented in red, as explained in the methods.

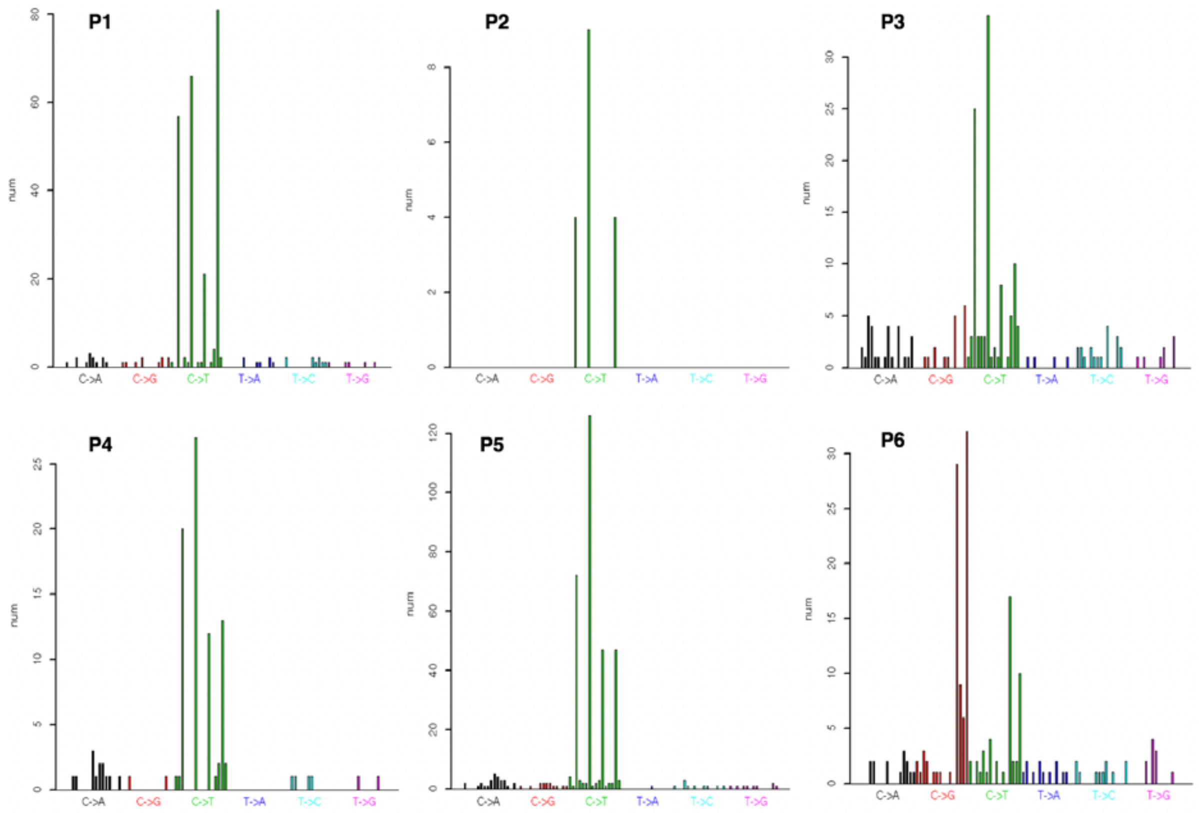

Supplementary Figure 2. The distribution of somatic mutations types for each patient. There are six classes of base substitutions, including C->A (black), C->G (red), C->T (green), T->A (blue), T->C (cyan), T->G (pink) (all substitutions are referred to by the pyrimidine of the mutated Watson-Crick base pair). Incorporating information on the bases immediately 5’ and 3’ to each mutated base results in 96 possible mutations types in this classification, represented in the x-axis. The number of each type of somatic mutation is represented by the y-axis. All patients show predominantly C > T transitions, similar to previous findings in somatic breast cancer mutations. Patient P6 shows both C >T and C > G transitions.

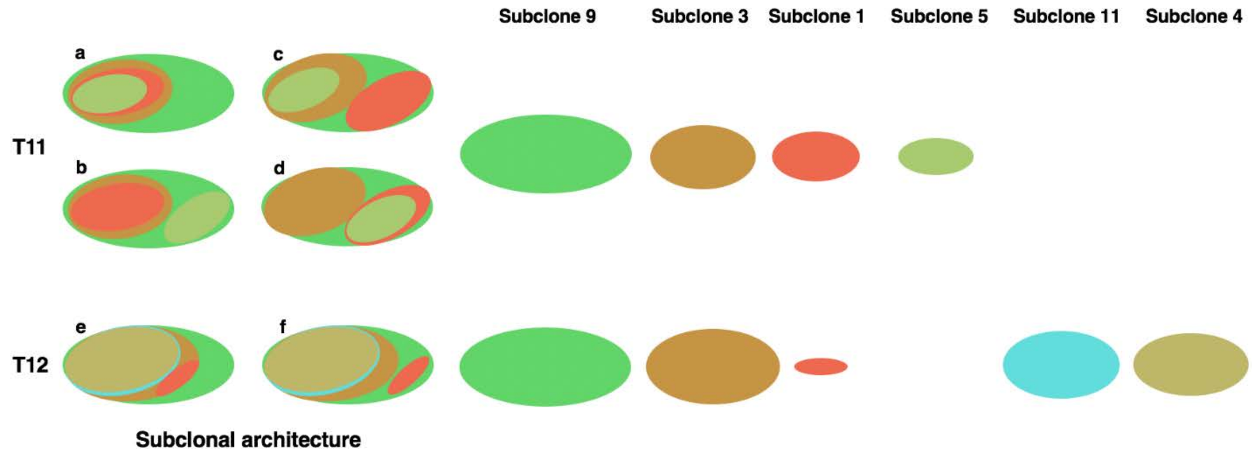

Supplementary Figure 3. Subclonal architecture for patient P6.
There are four different possible subclonal architectures for sample T11 (a, b, c and d) and two for sample T12 (e and f). If the subclonal architecture for T11 is a or b, then the corresponding subclonal architecture for T12 should be e, according to the deduced linear and/or branching relationships of these subclones. Similarly, if the subclonal architecture for T11 is c or d, then the corresponding subclonal architecture for T12 should be f. The area of the ovals is proportional to the estimated cluster CCF. Subclones are labelled in the same color as in Figure 3b. Subclone 9, 3 and 1 are shared by both samples in P6.

Supplementary Table 1. Clinical and pathological information of the six IBC patients

Patients	Race	Age	Sex	Sampling Date	TNM Stage	Grade	ER Positivity	PR Positivity	HER2 Status
**P1**	black	36	F	1993	T2, N1, M0	N/A	5%	80%	N/A
**P2**	N/A	52	F	1996	T4b, N1, M0	Nuclear 2-3 /Histologic 3	95%	40%	Score 3+
**P3**	white	62	F	1998	T3, N1b, M0	Nuclear 2 /Histologic 3	85%	25%	Score 0
**P4**	white	59	F	1999	T4b, N2, M0	Nuclear 2-3 /Histologic 3	80%	<2%	Score 2+
**P5**	white	72	F	2004	T2, N0, M0	N/A	>90%	>90%	Score 2+
**P6**	black	54	F	2012	T4d, N0, M1	Nuclear 3 /Histologic 3	95%	95%	Score 0

Note: *IBC* inflammatory breast cancer; *ER* estrogen receptor; *PR* progesterone receptor; *HER2* human epidermal growth factor receptor 2; *N/A* not available. The TNM staging system developed by the American Joint Committee on Cancer (AJCC) classifies cancers by the size and extent of the primary tumor (T), involvement of regional lymph nodes (N), and the presence or absence of distant metastases (M). Reporting results of HER2 testing by immunohistochemistry (IHC): Negative (Score 0); Negative (Score 1+); Equivocal (Score 2+); Positive (Score 3+).

Supplementary Table 2. Details of the tumor and normal tissue samples in the six IBC patients

Patients	Sample ID	Sample Type	Tissue Location	Sample Source	Somatic Mutations
**P1**	P1_N1	Normal	Upper outer quadrant of breast	Mastectomy	0
	P1_T1	Tumor	Areola		199
	P1_T2	Tumor	Tumor		94
**P2**	P2_N2	Normal	Not available	Incisional biopsy	0
	P2_T3	Tumor	Tumor		9
	P2_T4	Tumor	Remainder of breast		6
**P3**	P3_T5	Tumor	Tumor	Lumpectomy	142
	P3_T6	Tumor	Nipple		119
**P4**	P4_N3	Normal	Nipple	Mastectomy	0
	P4_T7	Tumor	Skin (tumor infiltration)		73
	P4_T8	Tumor	Tumor		47
**P5**	P5_T9	Tumor	Nipple in relation to tumor	Lumpectomy	233
	P5_T10	Tumor	Retroareolar tumor		262
**P6**	P6_N4	Normal	Lower inner quadrant of breast, part from T11's normal	Mastectomy	0
	P6_T11	Tumor	Tumor to nipple		161
	P6_T12	Tumor	Tumor		132

Note: *IBC* inflammatory breast cancer

Supplementary Table 3. QPLOT results, including mean depth, insert size and mapping rate

Patients	Sample ID	Mean depth	Insert size mode	Insert size medium	Mapping rate (%)	Map quality <10 (%)	Proper paired (%)	Mapped bases (e9)	Total reads (e6)	Q20 bases (e9)	Duplication rate (%)
**P1**	P1_N1	160.33	98	104	99.64	10.31	93.8	7.55	191.57	7.33	21.35
	P1_T1	174.3	109	118	99.48	9.39	91.29	8.21	194.28	7.93	13.89
	P1_T2	152.39	114	124	99.82	8.27	95.89	7.17	158.46	7.01	13.76
**P2**	P2_N2	162.53	118	127	99.24	9.75	89.07	7.65	186.98	7.34	12.26
	P2_T3	170.96	107	115	99.44	10.86	87.16	8.05	203.94	7.83	13.37
	P2_T4	161.66	112	121	99.32	9.53	91.07	7.61	193.65	7.39	16.99
**P3**	P3_T5	166.2	110	119	99.45	10.41	88.88	7.83	185.14	7.64	12.29
	P3_T6	142.06	115	123	99.26	11.15	90.22	6.69	160.29	6.39	10.6
**P4**	P4_N3	183.41	100	105	99.44	11.02	90.01	8.64	233.18	8.4	21.56
	P4_T7	133.33	91	94	99.38	10.44	94.06	6.27	173.97	6.12	25.87
	P4_T8	155.34	100	104	99.55	10.48	90.19	7.31	190.73	7.11	19.39
**P5**	P5_T9	209.93	106	115	99.49	6.93	94.94	9.88	210.2	9.57	13.32
	P5_T10	198.05	106	115	99.31	6.75	94.6	9.32	200.39	9.03	13.88
**P6**	P6_N4	176.33	129	141	99.9	6.78	97.3	8.29	177	8.12	11.38
	P6_T11	191.81	132	141	99.58	6.83	93.26	9.03	193.26	8.76	8.69
	P6_T12	187.01	116	132	99.3	6.48	97.1	8.8	193.84	8.41	13.11
