# Supplementary material for: Whole-exome sequencing identifies somatic mutations and intratumor heterogeneity in inflammatory breast cancer
Source: NPJ Breast Cancer. 2021 Jun 1;7:72. doi: 10.1038/s41523-021-00278-w (PMC8169683; doi:10.1038/s41523-021-00278-w)
Supplement: Supplementary file 3 — Reporting Summary [file 41523_2021_278_MOESM3_ESM.pdf]

nature research

Corresponding author(s): Hushan Yang

Last updated by author(s): Mar 10, 2021

Reporting Summary

Nature Research wishes to improve the reproducibility of the work that we publish. This form provides structure for consistency and transparency in reporting. For further information on Nature Research policies, see Authors & Referees and the Editorial Policy Checklist.

Statistics

For all statistical analyses, confirm that the following items are present in the figure legend, table legend, main text, or Methods section.

n/a	Confirmed	
☐	☒	The exact sample size (*n*) for each experimental group/condition, given as a discrete number and unit of measurement
☐	☒	A statement on whether measurements were taken from distinct samples or whether the same sample was measured repeatedly
☒	☐	The statistical test(s) used AND whether they are one- or two-sided<br>*Only common tests should be described solely by name; describe more complex techniques in the Methods section.*
☒	☐	A description of all covariates tested
☒	☐	A description of any assumptions or corrections, such as tests of normality and adjustment for multiple comparisons
☒	☐	A full description of the statistical parameters including central tendency (e.g. means) or other basic estimates (e.g. regression coefficient) AND variation (e.g. standard deviation) or associated estimates of uncertainty (e.g. confidence intervals)
☒	☐	For null hypothesis testing, the test statistic (e.g. *F*, *t*, *r*) with confidence intervals, effect sizes, degrees of freedom and *P* value noted<br>*Give P values as exact values whenever suitable.*
☒	☐	For Bayesian analysis, information on the choice of priors and Markov chain Monte Carlo settings
☒	☐	For hierarchical and complex designs, identification of the appropriate level for tests and full reporting of outcomes
☒	☐	Estimates of effect sizes (e.g. Cohen's *d*, Pearson's *r*), indicating how they were calculated

Our web collection on statistics for biologists contains articles on many of the points above.

Software and code

Policy information about availability of computer code

Data collection	No software was used for data collection.
Data analysis	All of the software used and the version numbers have been stated in the Methods section. The software used for data analysis include BWA v0.7.17, samtools v1.9, GATK v4.1.0.0, QPLOT, MuTect2 and ANNOVAR. HMMcopy v0.99.0 and TITAN v1.26.0 were used to infer CNA and PyClone v0.13.1 was used to infer subclone.

For manuscripts utilizing custom algorithms or software that are central to the research but not yet described in published literature, software must be made available to editors/reviewers. We strongly encourage code deposition in a community repository (e.g. GitHub). See the Nature Research guidelines for submitting code & software for further information.

Data

Policy information about availability of data

All manuscripts must include a data availability statement. This statement should provide the following information, where applicable:

- Accession codes, unique identifiers, or web links for publicly available datasets
- A list of figures that have associated raw data
- A description of any restrictions on data availability

A full data availability statement is included in the manuscript.

Field-specific reporting

Please select the one below that is the best fit for your research. If you are not sure, read the appropriate sections before making your selection.

☒ Life sciences ☐ Behavioural & social sciences ☐ Ecological, evolutionary & environmental sciences

For a reference copy of the document with all sections, see nature.com/documents/nr-reporting-summary-flat.pdf

Life sciences study design

All studies must disclose on these points even when the disclosure is negative.

Sample size	Sample size: N=6. We sequenced all of the samples we could obtain. There was no sample size calculation.
Data exclusions	We excluded the following subjects from this study, including those patients who were co-infected by other virus such as HCV and HIV, or had other malignacies.
Replication	This is a sequencing study without data from in vivo/in vitro experiments.
Randomization	This is not a clinical trial. The randomization of participants was not relevant to this study.
Blinding	The persons conducted the sequencing experiments were blind to the status of the participants.

Reporting for specific materials, systems and methods

We require information from authors about some types of materials, experimental systems and methods used in many studies. Here, indicate whether each material, system or method listed is relevant to your study. If you are not sure if a list item applies to your research, read the appropriate section before selecting a response.

Materials & experimental systems

n/a	Involved in the study	
☒	☐	Antibodies
☒	☐	Eukaryotic cell lines
☒	☐	Palaeontology
☒	☐	Animals and other organisms
☐	☒	Human research participants
☒	☐	Clinical data

Methods

n/a	Involved in the study	
☒	☐	ChIP-seq
☒	☐	Flow cytometry
☒	☐	MRI-based neuroimaging

Human research participants

Policy information about studies involving human research participants

Population characteristics	The participants' demographic and clinical characteristics including age, gender, hormone receptors status, HER2 status, tumor grade were collected.
Recruitment	Patients in the discovery cohort were recruited from Thomas Jefferson University Hospital from 1993 to 2012. All the patients were IBC patients. Therefore, the findings in this study may not be generalized to the population of other ethnic origin.
Ethics oversight	This study was approved by the Institutional Review Board of Thomas Jefferson University.

Note that full information on the approval of the study protocol must also be provided in the manuscript.
